# Andrographolide Suppresses Pyroptosis in *Mycobacterium tuberculosis-Infected* Macrophages via the microRNA-155/Nrf2 Axis

**DOI:** 10.1155/2022/1885066

**Published:** 2022-04-28

**Authors:** Yan Fu, Jingjing Shen, Fanglin Liu, Hemin Zhang, Yuejuan Zheng, Xin Jiang

**Affiliations:** ^1^Center for Traditional Chinese Medicine and Immunology Research, School of Basic Medical Sciences, Shanghai University of Traditional Chinese Medicine, Shanghai 201203, China; ^2^The Research Center for Traditional Chinese Medicine, Shanghai Institute for Major Infectious Diseases and Biosafety, Shanghai 200032, China; ^3^Shanghai Key Laboratory of Health Identification and Assessment, Shanghai University of Traditional Chinese Medicine, Shanghai 201203, China

## Abstract

Tuberculosis (TB) remains a leading threat to public health worldwide with *Mycobacterium tuberculosis* (Mtb) infections causing long-term abnormal and excessive inflammatory responses, which in turn lead to lung damage and fibrosis, and ultimately death. Host-directed therapy (HDT) has been shown to be an effective anti-TB strategy in the absence of effective anti-TB drugs. Here, we used an *in vitro* macrophage model of Mtb infection to evaluate the effects of andrographolide (Andro), extracted from *Andrographis paniculata*, on pyroptosis in Mtb-infected macrophages. We evaluated the molecular mechanisms underlying these outcomes. These evaluations revealed that Andro downregulated the expression of proinflammatory miR-155-5p, which then promoted the expression of Nrf2 to suppress pyroptosis in Mtb-infected macrophages. Further study also demonstrated that siNrf2 could attenuate the inhibitory effect of Andro on TXNIP, validating our mechanistic studies. Thus, our data suggest that Andro may be a potential candidate adjuvant drug for anti-TB therapy as it inhibits pyroptosis in Mtb-infected macrophages, potentially improving clinical outcomes.

## 1. Introduction

Despite extensive intervention, tuberculosis (TB) remains one of the most lethal infectious diseases in the world, and although there are several first-line drugs for the treatment of TB, they generally have several shortcomings, including long treatment cycles and major side effects. Moreover, the severe inflammatory damage resulting from active TB infection can also reduce the treatment efficiency. The last several decades have witnessed a substantial effort to develop novel anti-TB drugs, but the outcomes of these studies remain unsatisfactory. The major problems associated with this development include slow progress in developing anti-TB drugs and inevitable drug resistance of TB [1]. Therefore, a novel strategy for treating TB is urgently required.

Current researches suggest that most TB patients do not die directly from *Mycobacterium tuberculosis* (Mtb) infection, but rather from the tissue damage induced by the long-term excessive inflammatory response associated with these infections [2, 3]. Moreover, excessive inflammatory responses also promote the spread of Mtb and reduce treatment efficacy [4]. This implies that avoiding the excessive immune response of the body can reduce the risk of death in TB patients and enhance the efficacy of anti-TB drugs, making it a novel target for interventional strategies.

Host-directed therapy (HDT) is a novel adjuvant therapy against TB which is designed to use drugs to regulate the protective immune response against the pathogen and reduce excessive inflammation in the infected tissues [5]. HDT drugs can help the host to reduce the excessive inflammatory response in Mtb-infected macrophages, which act as the primary effector cells for the elimination of Mtb [6], thus alleviating tissue damage and improving the treatment efficiency of the anti-TB drugs [7].

Pyroptosis is a proinflammatory form of programmed cell death and has been shown to induce excessive inflammatory responses in affected tissues resulting in serious tissue damage and pathological progression [8]. In TB, pyroptosis in Mtb-infected macrophages not only releases several pro-inflammatory mediators, including high mobility group protein (HMGB1) and interleukin-1beta (IL-1*β*), but also spreads the Mtb bacteria, aggravating the pathologic process and increasing the risk of infection [9–11].

microRNAs (miRNAs) are noncoding transcripts of 18-25 nucleotides, which interact with the 3′-untranslated regions (3′-UTRs) of their targets mRNAs resulting in their degradation or inhibition of their translation [12, 13]. Since the initial discovery of their regulatory role in the development of the nematodes, miRNAs have emerged as key modulators in many biological processes, including the inflammatory response [14, 15]. Studies have revealed that there are a variety of key miRNAs associated with infection and disease progression. One example is miR-155-5p which acts as a proinflammatory miRNA participating in several infectious diseases [16, 17]. Furthermore, research also has shown that miR-155-5p is upregulated in macrophages infected with Mtb [18, 19]. It has also been demonstrated that multiple miRNAs can modulate the same target, and a single miRNA can modulate multiple targets [20, 21]. In addition, studies have demonstrated that miR-155-5p regulates several genes, including suppressor of cytokines signaling1 (SOCS1), silent information regulator 1 (Sirt1), autophagy-related gene 12 (ATG12), and nuclear factor erythroid 2-related factor 2 (Nrf2) [22–25], which might ameliorate the inflammatory response [26–29].

Nrf2 is an essential regulator of the antioxidant response [30, 31]. Oxidative stress stabilizes Nrf2 protein expression and initiates the activation of a multistep pathway that includes its nuclear translocation, recruitment of transcriptional coactivators, and subsequent binding of antioxidant response elements within its target genes [32, 33]. Several studies have revealed that activation of the Nrf2/HO-1 signaling pathway could contribute to the inhibition of oxidative stress induced pyroptosis [34], while other studies have shown that it can also attenuate pyroptosis by suppressing the activation of the NLRP3 inflammasome [35, 36].

Andrographolide (Andro) is the major bioactive component of *Andrographis paniculata*, which is known to exert several biological effects, including anti-inflammatory, antitumor, antivirus, and antioxidant effects [37–40]. Moreover, treatment with Andro can activate Nrf2 promoting HO-1 expression and suppressing oxidative stress and neuroinflammation in primary astrocytes [41]. Our previous study has also revealed that Andro treatment can inhibit the inflammatory response in Mtb-infected macrophages [42]. Given that traditional Chinese medicines generally have applications against multiple targets, we speculate that Andro could also inhibit the inflammatory response using other pathways. Moreover, recent publications have revealed that Andro treatment suppresses pyroptosis via inhibiting the activation of the AIM2 inflammasome [37]. However, to our knowledge, there is no relevant report discussing whether Andro treatment might suppress macrophage pyroptosis via the miR-155/Nrf2 axis. Here, we describe a novel mechanism of Andro and demonstrate that it could be used as an adjuvant drug against TB to alleviate excessive inflammatory response via suppression of Mtb-infected macrophage pyroptosis.

## 2. Materials and Methods

### 2.1. Chemicals and Reagents

Western and IP lysis buffer, BCA protein assay kit, protein A/G agarose/sepharose beads, and propidium iodide (PI) were bought from the Beyotime Institute of Biotechnology (Shanghai, China). The following antibodies were used: anti-Nrf2 (12721), anti-HO-1 (43966), and anti-NLRP3 (15101S) were bought from Cell Signaling Technology, Inc.; anti-GSDMD (A20197), anti-Histone3 (A2348), and goat anti-NLRP3 (A14223) were obtained from ABclonal Technology (Wuhan, China); and anti-TXNIP (sc-166234) and anti-TRX2 (sc-133201) were obtained from Santa Cruz Biotechnology, Inc. Middlebrook 7H9 and 7H10 media were purchased from Difco (Detroit, MI, USA); oleic acid-albumin-dextrose-catalase (OADC) supplements were obtained from BD Biosciences (BD, Sparks, MD, USA). RiboFECT CP transfection kit, mouse miR-155-5p mimic (miR1CM001), mouse mimic NC #22 (miR1N0000001-1-5), mouse miR-155-5p inhibitor (miR20000165-1-5), mouse inhibitor NC #24 (miR2N0000002-1-5), mouse miR-155-5p antagomir (miR31148164306-4-5), and mouse antagomir NC #24 (miR3N0000002-4-5) were obtained from Ribo Biotechnology (Guangzhou, China). IL-1*β* ELISA kit was bought from R&D (USA). NE-PER™ nuclear and cytoplasmic extraction reagent kit (78833) and SuperSignal™ West Femto maximum sensitivity substrate (34096) were bought from Thermo Fisher Scientific (USA). EZ-press RNA purification kit was obtained from EZBioscience. PrimeScript™ RT reagent kit (RR037A and RR036A) and SYBR RT-PCR kits were purchased from Takara Shuzo, Otsu (Japan). RT-PCR primers were purchased from Sangon Biotech (Shanghai, China).

### 2.2. Animals

Male C57BL/6J mice (6-8 weeks, 20 ± 3 g) were obtained from Vital River Laboratory Animal Technology Co., Ltd. (Beijing, China). Animal experiments strictly follow the National Institute of Health Guide for the Care and Use of Laboratory Animals, with the approval of the Scientific Investigation Board of Shanghai University of Traditional Chinese Medicine (Shanghai, China).

### 2.3. Drugs

Andrographolide (Andro) (molecular weight: 350.45, purity >98%) is isolated from *Andrographis paniculate* (the full names of drug have been checked according to http://www.theplantlist.org/). Andro was obtained from Shanghai Tauto Biotech Co., Ltd. (CAS:5508-58-7) (Shanghai, China).

### 2.4. Cell Culture

Mouse macrophage-like cell line Raw264.7 was obtained from ATCC (Manassas, VA) and cultured in DMEM supplemented with 10% fetal bovine serum (FBS) in 5% CO_2_ at 37°C as described previously [43]. Thioglycolate-elicited mouse primary peritoneal macrophages were prepared from male C57BL/6J mice (6-8 weeks of age) as described previously [44].

### 2.5. Bacterial Strains

The Mtb H37Ra was used in this study. H37Ra strain was grown in Middlebrook 7H9 or 7H10 broth supplemented with 0.2% glycerol, 0.05% Tween-80, and 10% Middlebrook OADC supplement.

### 2.6. Mtb Infection

The cells were seeded at the appropriate cell culture plates and grown at 37°C overnight. The cells were infected with Mtb H37Ra at a MOI (multiplicity of infection) of 10 : 1. After 4 h of co-incubation at 37°C, cells were washed three times with sterile phosphate-buffered saline (PBS) and cultured with DMEM containing 10% FBS in the presence and absence of Andro (25 *μ*M) for different times as described previously [43].

### 2.7. Western Blotting

The cells were lysed in lysis buffer after incubation with or without Andro (25 *μ*M) for different times (6, 12, and 24 hours). Then, BCA protein assay kit was used to determine the total protein concentration of each sample. Then, 5 × SDS loading buffer was added to the whole cell lysate followed by boiled 10 min, and then, the proteins were separated by SDS-PAGE and transferred onto nitrocellulose membranes followed by blocking with 5% (w/v) bovine serum albumin and incubated with specific primary antibodies at 4°C overnight. Then, the membranes were washed with TBST for three times followed by incubation with HRP-conjugated secondary antibodies at room temperature for 1 hour. Chemiluminescence was tested with ECL-chemiluminescent kit (Thermo Scientific). The quantification of each protein was performed by Image J-1.51(100) (2015) as described previously [43].

### 2.8. RNA Quantification

The Raw264.7 cells or primary peritoneal macrophage cells were seeded at 6-well cell culture plates and grown at 37°C overnight. The cells were infected with Mtb H37Ra (MOI = 10 : 1). Then, the cells were washed three times with sterile PBS after 4 hours and cultured with DMEM containing 10% FBS in the presence and absence of Andro (25 *μ*M) for 6, 12, and 24 hours. The total RNA, including miRNA, was extracted with EZ-press RNA purification kit according to the manufacturer's instruction. The miRNA and mRNAs were reverse transcribed using PrimeScript™ RT reagent kit (Takara, RR037A) and PrimeScript™ RT reagent kit (Takara, RR036A), respectively. Real-time quantitative RT-PCR analysis was performed by the SYBR RT-PCR kits. The relative expression level of miR-155-5p and mRNAs was normalized to that of internal control U6 and *β*-actin, respectively, by using a 2^−∆∆Ct^ cycle threshold method. cDNA from various cell samples was amplified with specific primers (Mouse-miR-155-5p: Forward 5′-GCGCGTTAATGCTAATTGTGAT-3′ and Reverse 5′-AGTGCAGGGTCCGAG GTATT-3′, RT 5′-GTCGTATCCAGTGCAGGGTCCGAGGTATTCGCACTGGAT ACGACACCCCT-3′; Mouse-U6: Forward 5′-GCTTCGGCAGCACATATACTAA AAT-3′ and Reverse 5′-CGCTTCACGAATTTGCGTGTCAT-3′, RT5′-CGCTTCAC GAATTTGCGTGTCAT-3′; Mouse-miR-155HG: Forward 5′-TTTGGCCTCTGACT GACTCCTACC-3′ and Reverse 5′-GTTCATCCAGCAGGGTGACTCTTG-3′; Mouse-Nrf2: Forward 5′-CAGCATAGACAGGACATGGAG-3′ and Reverse 5′-GAA CAGCGGTAGTATCAGCCAG-3′; Mouse-HO-1: Forward 5′-CACTCTGGAGATG ACACCTGAG-3′ and Reverse 5′-GTGTTCCTCTGTCAGCATCACC-3′; Mouse-NQO-1: Forward 5′-GCCGAACACAAGAAGCTGGAAG-3′ and Reverse 5′-GGCAAATCCTGCTACGAGCACT-3′; Mouse-*β*-actin: Forward 5′-CATTGCTGA CAGGATGCAGAAGG-3′ and Reverse 5′-TGCTGGAAGGTGGACAGTGAGG-3′).

### 2.9. Coimmunoprecipitation

The cells were seeded at 35 mm dishes and infected with Mtb H37Ra (MOI = 10 : 1) after adherence. Then, PBS was used to washed the cells for three times after 4 hours and followed by cultured with or without Andro (25 *μ*M) for 12 hours. The cells were lysed by lysis buffer and centrifugated (12000 rpm, 10 min). Samples containing equal amounts of protein were incubated with specific antibodies overnight at 4°C with gentle rotation. Then, the samples were added with protein A/G agarose/sepharose beads at 4°C with gentle rotation 4 hours followed by washing with lysis buffer four times. The samples were boiled in 1 × SDS buffer before SDS-PAGE electrophoresis. The steps after SDS-PAGE electrophoresis are the same as western blotting measurement as described previously [43].

### 2.10. Immunofluorescence

The cells were seeded at culture plates and grown at 37°C overnight. Then, the cells were infected with Mtb H37Ra (MOI = 10 : 1). Then, the cells were washed three times with sterile PBS after 4 hours and cultured with DMEM containing 10% FBS in the presence and absence of Andro (25 *μ*M) for 12 hours. The cells were washed with PBS for three times and fixed with 4% paraformaldehyde at room temperature for 15 min. The fixed cells were washed three times with PBS and permeabilized with 2% of Triton X-100 for 15 min at room temperature. Then, the cells were blocked with 5% bovine serum albumin for 0.5 hours at room temperature followed by staining with primary antibodies 4°C overnight. Then, the cells were washed three times with PBS followed by incubation with secondary antibodies for 2 hours. Then, the cells were washed with PBS for three times followed by staining with DAPI for 10 min. Fluorescence images were acquired on the Zeiss confocal microscopy system as described previously [43].

### 2.11. LDH Release Assay

The lactate dehydrogenase (LDH) cytotoxicity assay kit was used to assess the cell death level. The cells were seeded at 24-well plates and grown at 37°C overnight. Then, Mtb H37Ra (MOI = 10 : 1) was used to infect the cells. After incubation for 4 hours, the cells were washed with PBS for three times followed by addition with or without Andro (25 *μ*M) medium. The cell supernatant was collected and centrifuged (1000 rpm, 5 min) after incubation appropriate times. Then, the LDH levels were determined according to the manufacturer's protocol as described previously [43].

### 2.12. IL-1*β* ELISA

The cells were seeded at 24-well plates and grown at 37°C overnight. Then Mtb H37Ra (MOI = 10 : 1) was used to infect the cells. After incubation 4 hours, the cells were washed with PBS with three times and followed by addition with or without Andro (25 *μ*M) medium. Then, the supernatants of the cells were collected and applied enzyme-linked immunosorbent assay (ELISA) kits to assess the level of IL-1*β* according to the manufacturer's protocol as described previously [43].

### 2.13. PI Staining

The cells were infected with Mtb H37Ra (MOI = 10 : 1), washed with PBS for three times after 4 hours incubation, and followed by addition with or without Andro (25 *μ*M) medium.

Then, the cells were collected and followed by staining with 2 *μ*g/ml propidium iodide (PI) for 10 min. Cell death rates were measured by C6 flow cytometer (BD Accuri™) as described previously [43].

### 2.14. RNA Interference

The cells were transfected with 20 *μ*M siRNA, 50 *μ*M miR-155-5p mimic, miR-155-5p inhibitor, or 100 *μ*M miR-155-5p antagomir for 24 hours. Then, the cells were infected with Mtb H37Ra (MOI = 10 : 1) and washed with PBS for three times followed by addition with or without Andro (25 *μ*M) medium after 4 hours. Then, the cells were collected after incubation for appropriate times followed by western blotting or RT-PCR analysis. The siRNA sequences are as follows:

Nrf2-mus-931 (KD1) sense 5′-GCAACUGUGGUCCACAUUUTT-3′,

Antisense 5′-AAAUGUGGACCACAGUUGCTT-3′;

Nrf2-mus-793 (KD2) sense 5′-CCGAAUUACAGUGUCUUAATT-3′,

Antisense 5′-UUAAGACACUGUAAUUCGGTT-3′;

Nrf2-mus-275 (KD3) sense 5′-GCAGGACAUGGAUUUGAUUTT-3′,

Antisense 5′-AAUCAAAUCCAUGUCCUGCTT-3′;

Negative control sense 5′-UUCUCCGAACGUGUCACGUTT-3′,

Antisense 5′-ACGUGACACGUUCGUAGAATT-3′.

The mus-miR-155-5p mimic was 5′-UUAAUGCUAAUUGUGAUAGGGGU-3′.

### 2.15. Statistical Analysis

GraphPad Prism 8 (GraphPad Software, La Jolla, CA, USA) was used to statistical analysis. Statistical significance was performed using one-way ANOVA analysis, and results were given as the means ± standard deviation (SD). Data shown are representative of at least triplicate experiments. *p* < 0.05 was considered to be statistically significant.

## 3. Results

### 3.1. Andro Ameliorates Pyroptosis in Mtb-Infected Macrophages

**Figure 1:**
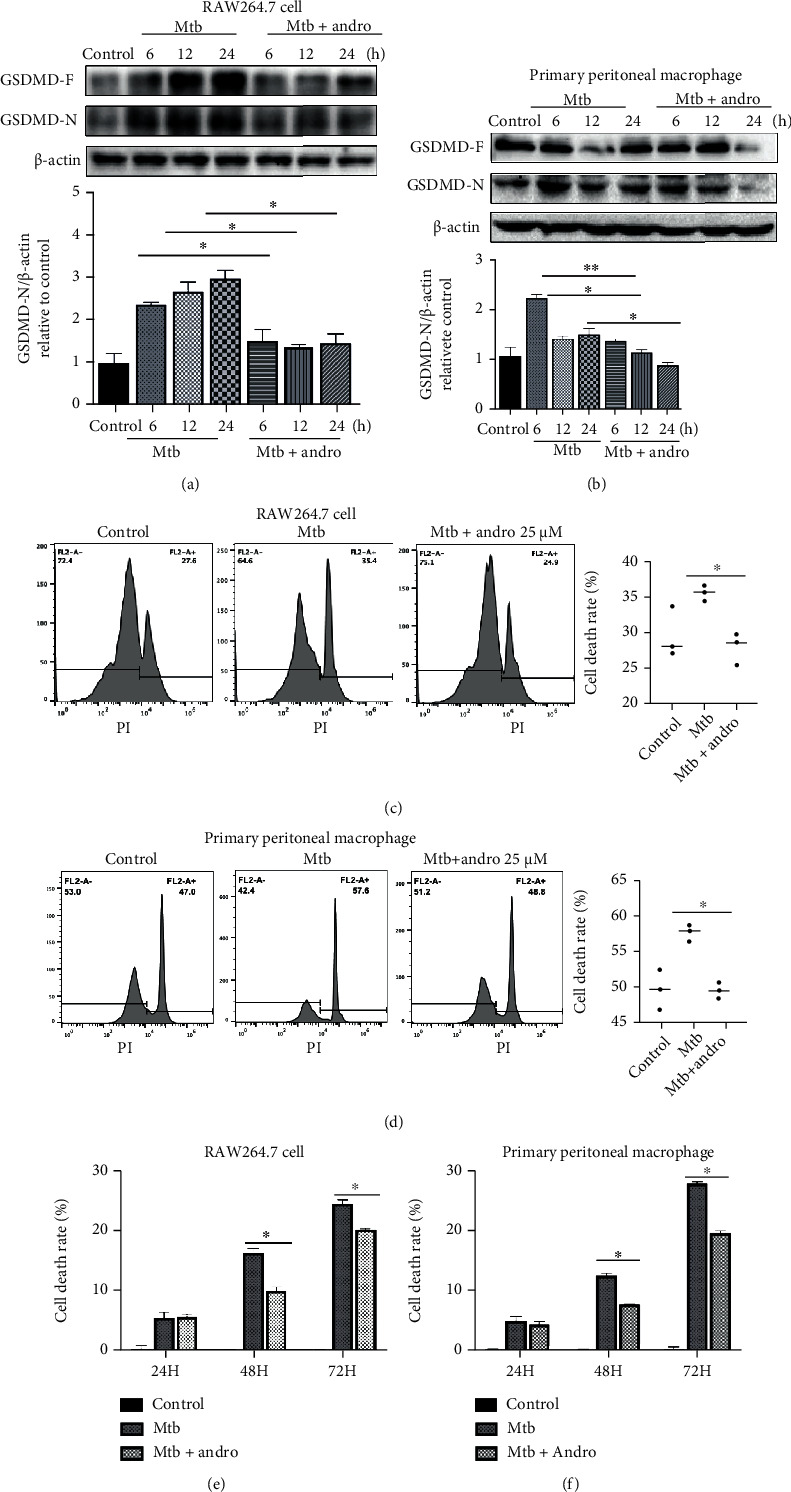
Andro ameliorates pyroptosis in Mtb-infected macrophages. (a, b) Levels of GSDMD-N protein were detected by western blotting in RAW264.7 cells and primary peritoneal macrophage cells, respectively. (c, d) The viable and dead cell populations were measured by PI staining in RAW264.7 cells and primary peritoneal macrophage cells, respectively. FL2-A+ represents the dead cell populations; FL2-A- represents the viable cell populations. (e, f) Cell death was detected by using the lactate dehydrogenase (LDH) cytotoxicity assay kit in RAW264.7 cells and in primary peritoneal macrophage cells, respectively. Data are shown as mean ± SD of three independent experiments. ∗*p* < 0.05, ∗∗*p* < 0.01.

Given the role of pyroptosis in inflammation, we hypothesized that Andro may reduce inflammation in TB by suppressing pyroptosis in Mtb-infected macrophages. Here, we used Mtb to infect RAW264.7 cells and primary peritoneal macrophage cells to produce an appropriate set of experimental models. According to a previous MTT measurement [42], we used 25 *μ*M Andro to treat Mtb-infected macrophages and then assessed GSDMD-N levels using western blotting. The results showed that Mtb infection induced the cleavage of GSDMD, while Andro treatment significantly downregulated the expression of GSDMD-N (Figures 1(a) and 1(b)).

We also used propidium iodide (PI) staining and flow cytometric assay to determine whether Andro treatment affected the viability of macrophages and demonstrated that Mtb increased the death rate in infected macrophages, while Andro treatment significantly reduced this effect (Figures 1(c) and 1(d)).

Lactate dehydrogenase (LDH) is a relatively stable enzyme released during cell death and is often used as an adjuvant marker of pyroptosis [45]. Based on this, we evaluated LDH levels in these cells in an effort to assess the level of pyroptosis. Both macrophage models revealed that Mtb infection induced increased cell death over time, while Andro treatment reduced this trend (Figures 1(e) and 1(f)). This is consistent with our previous findings, which demonstrated that Andro treatment of Mtb-infected macrophages reduces IL-1*β* level, suggesting a decrease in proinflammatory cytokines. Taken together, these results indicate that Andro treatment ameliorates pyroptosis in Mtb-infected macrophages.

### 3.2. Andro Inhibits Pyroptosis in Mtb-Infected Macrophages via Suppression of miR-155HG/miR-155-5p

**Figure 2:**
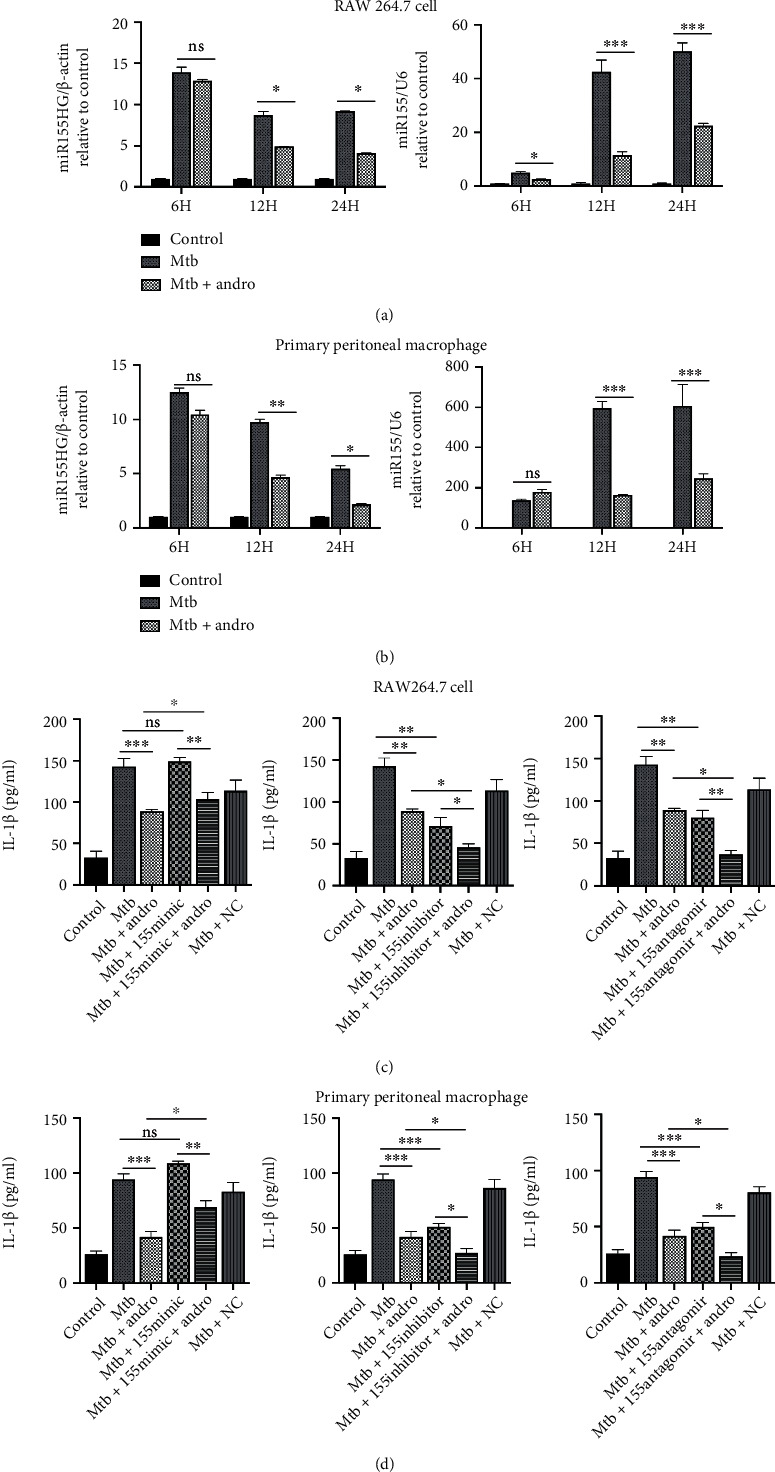
Andro inhibits pyroptosis in Mtb-infected macrophages through suppressing miR-155HG/miR-155-5p. (a, b) The expression of miR-155HG and miR-155-5p was analyzed by RT-PCR in RAW264.7 cells and primary peritoneal macrophage cells, respectively. (c, d) Levels of IL-1*β* determined by ELISA measurement in macrophage cells and primary peritoneal macrophage cells, respectively. Data are shown as mean ± SD of three independent experiments. NC: negative control. ∗*p* < 0.05, ∗∗*p* < 0.01, ∗∗∗*p* < 0.001. ns: nonsignificant.

Several researches have demonstrated that there are many miRNAs implicated in excessive inflammatory response and pyroptosis [46–49], including miR-155-5p [18]. Some of these studies have shown that miR-155-5p promotes macrophage pyroptosis via regulation of the NLRP3 inflammasome [50]. Thus, we hypothesized that miR-155-5p may be a key target in the Andro-mediated inhibition of pyroptosis in Mtb-infected macrophages. Thus, we decided to evaluate the expression of miR-155-5p in response to both Mtb and Andro treatment using RT-PCR. These evaluations showed that miR-155-5p was upregulated in response to increasing time of infection and that this was significantly reduced following the addition of Andro, in both macrophage models (Figures 2(a) and 2(b)).

MiR-155HG is a precursor of miR-155-5p, which also acts as a long noncoding RNA (lncRNA) regulating M1/M2 macrophage polarization in chronic obstructive pulmonary disease [51–53]. We also evaluated the expression of miR-155HG under these treatment conditions, with these experiments producing similar results to miR-155-5p. Mtb infection increases the expression of miR-155HG, whereas Andro treatment reduces its expression in response to Mtb (Figures 2(a) and 2(b)).

To further confirm whether Andro inhibits pyroptosis in Mtb-infected macrophages through the downregulation of miR-155-5p, we assessed GSDMD-N and IL-1*β* using miR-155-5p mimic, inhibitor, and antagomir.

**Figure 3:**
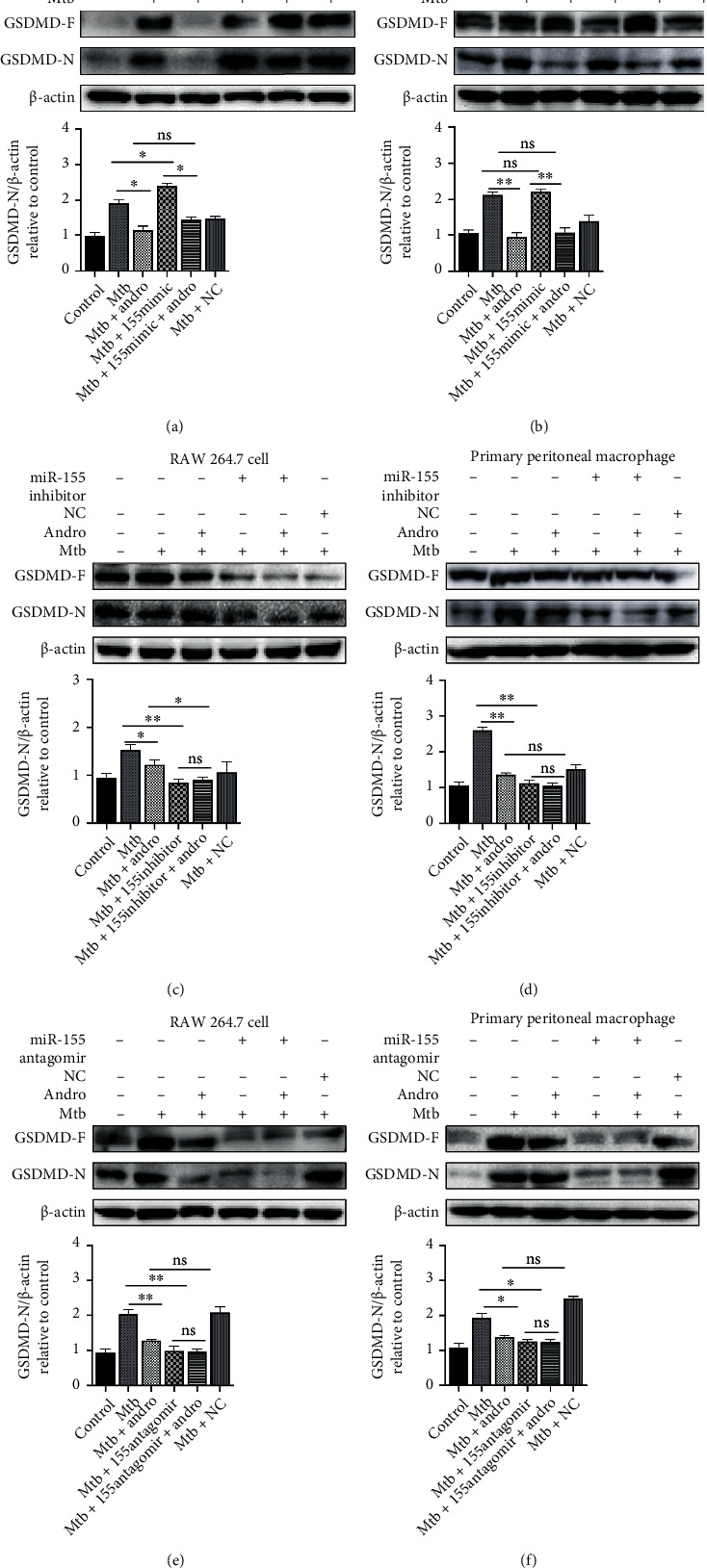
Andro inhibits pyroptosis in Mtb-infected macrophages through suppressing miR-155HG/miR-155-5p. (a, b) Levels of GSDMD-N were assessed by western blotting after miR-155-5p mimic transfection in RAW264.7 cells and primary peritoneal macrophage cells, respectively. (c, d) The expression of GSDMD-N was investigated by western blotting after miR-155-5p inhibitor transfection in RAW264.7 cells and primary peritoneal macrophage cells, respectively. (e, f) The expression of GSDMD-N was investigated by western blotting after miR-155-5p antagomir transfection in RAW264.7 cells and primary peritoneal macrophage cells, respectively. Data are shown as mean ± SD of three independent experiments. NC: negative control. ∗*p* < 0.05, ∗∗*p* < 0.01. ns: nonsignificant.

Interestingly, the overexpression of intracellular miR-155-5p increased Mtb-induced IL-1*β* expression and acted as an antagonist of the Andro-mediated downregulation of this cytokine, suggesting that an increase in miR-155-5p expression may exacerbate inflammation and impair the anti-inflammatory effects of Andro (Figures 2(c) and 2(d)). Similarly, GSDMD-N in Mtb-infected macrophages was also slightly upregulated in response to treatment with miR-155-5p mimic, while the anti-inflammatory effects of Andro were inhibited by its overexpression, suggesting that miR-155-5p expression promotes pyroptosis and Andro treatment inhibits it (Figures 3(a) and 3(b)). Furthermore, both miR-155-5p inhibitor and antagomir produced similar effects to the Andro treatment, including a reduction in IL-1*β* (Figures 2(c) and 2(d)) and GSDMD-N (Figures 3(c)–3(f)), suggesting an overall decrease in pyroptosis. Taken together, these results suggest that Andro inhibits pyroptosis in Mtb-infected macrophages via the downregulation of miR-155-5p, which may reduce the overall inflammatory response and pyroptosis in Mtb-infected macrophages.

Notably, IL-1*β* was further downregulated, and infected cells were treated with both miR-155-5p inhibitor or antagomir and Andro, suggesting that there are likely other mechanisms underlying Andro-mediated reductions in the inflammatory response. However, this phenomenon was not observed in evaluations of GSDMD-N (Figures 3(c)–3(f)).

### 3.3. Andro Activates the Nrf2/HO-1 Pathway through Inhibition of miR-155-5p Expression in Mtb-Infected Macrophages

**Figure 4:**
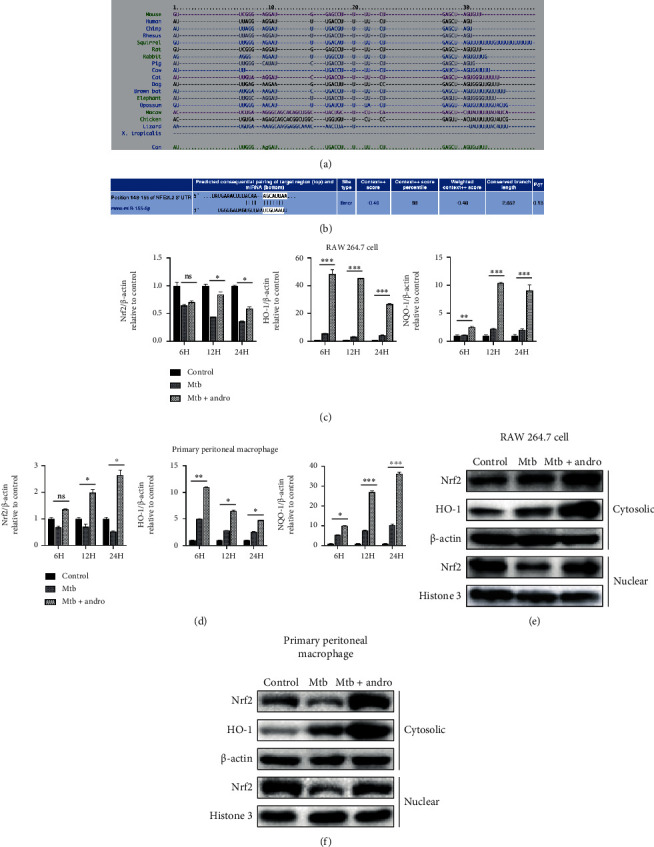
Andro activates the Nrf2/HO-1 pathway in Mtb-infected macrophages. (a) MiR-155-5p sequences in different species. (b) The target site of miR-155-5p on Nrf2 mRNA in mice. (c, d) The levels of Nrf2, HO-1, and NQO-1 mRNA were investigated by RT-PCR in RAW264.7 cells and primary peritoneal macrophage cells, respectively. (e, f) Total cytosolic and nuclear proteins were extracted to detect Nrf2 expression followed by western blotting using an anti-Nrf2 antibody in RAW264.7 cells and primary peritoneal macrophage cells, respectively. Data are shown as mean ± SD of three independent experiments. ∗*p* < 0.05, ∗∗*p* < 0.01, ∗∗∗*p* < 0.001. ns: nonsignificant.

MiRNAs generally work by binding to and regulating the expression of downstream target genes. Based on this, we evaluated the mechanism underlying these anti-inflammatory effects by identifying and evaluating the potential targets of miR-155-5p. We used TargetScan (http://www.targetscan.org) and found that the 3′-UTR of mouse Nrf2 mRNA as a putative target site for miR-155-5p (Figures 4(a) and 4(b)).

Studies have shown that Andro can inhibit inflammation by regulating the expression of Nrf2 [54, 55]. To determine whether Andro has an effect on Nrf2 and its downstream antioxidant genes in Mtb infection model, the mRNA of Nrf2, HO-1, and NQO-1 (HO-1 and NQO-1 are the downstream antioxidant genes of Nrf2) was detected in Mtb group and Andro group.

The results suggested that Andro significantly upregulated the expressions of these antioxidant genes (Figures 4(c) and 4(d)). Generally, accumulation of Nrf2 and its translocation into the nucleus can promote the expression of HO-1 and NQO-1. Thus, we also analyzed the protein levels of Nrf2 in the cytoplasm and nucleus of these cells and demonstrated that Andro treatment induced nuclear translocation of Nrf2 and increased the expression of HO-1 in the cytoplasm (Figures 4(e) and 4(f)).

**Figure 5:**
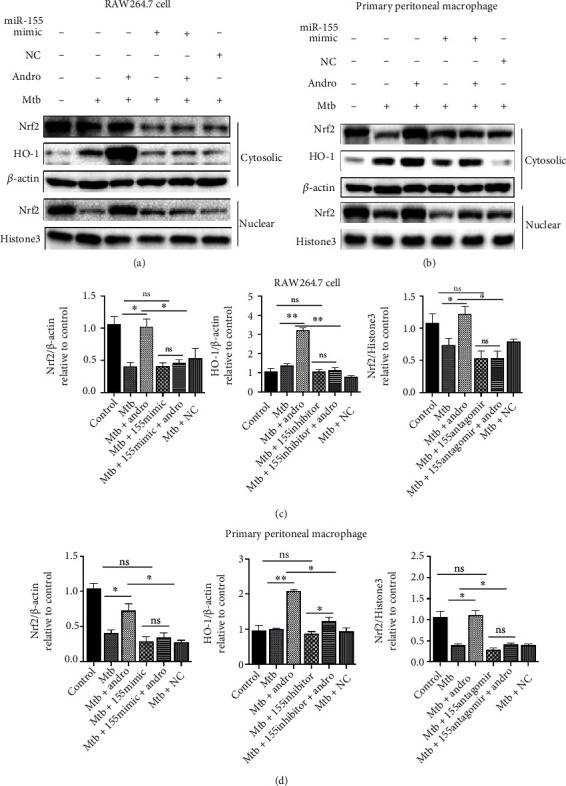
miR-155-5p mimic downregulates the expression of Nrf2 and HO-1 in Mtb-infected macrophages. (a, c) The expressions of Nrf2 and HO-1 were analyzed by western blotting after miR-155-5p mimic treatment in RAW264.7 cells. (b, d) The levels of Nrf2 and HO-1 proteins were assessed by western blotting after miR-155-5p mimic treatment in primary peritoneal macrophage cells. Data are shown as mean ± SD of three independent experiments. NC: negative control. ∗*p* < 0.05, ∗∗*p* < 0.01. ns: nonsignificant.

To determine whether Nrf2 is regulated by miR-155-5p, we assessed the levels of Nrf2 in the cytoplasm and nucleus of cells treated with miR-155-5p mimic. In the miR-155-5p mimic group, the mimic weakened the translocation of Nrf2, and the level of HO-1 induced by Andro (Figures 5(a)–5(d)) revealed that miR-155-5p is a mediator of Nrf2 and HO-1.

**Figure 6:**
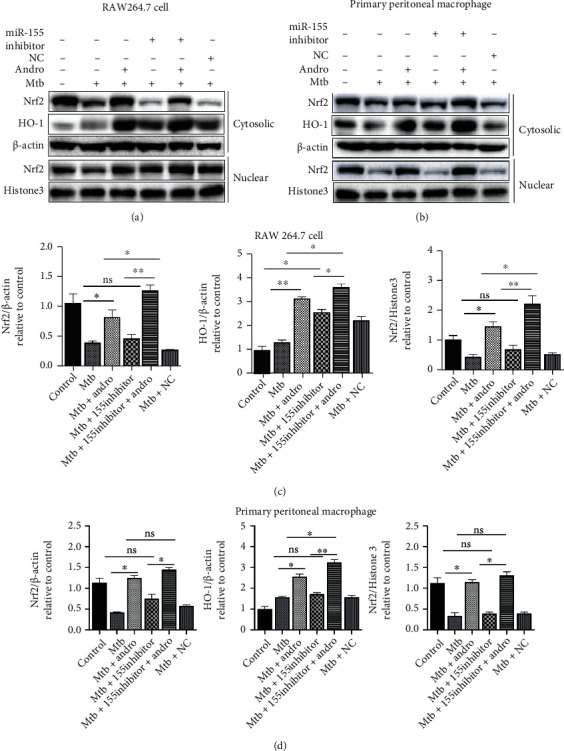
miR-155-5p inhibitor upregulates the expression of Nrf2 and HO-1 in Mtb-infected macrophages. (a, c) The expressions of Nrf2 and HO-1 were analyzed by western blotting after miR-155-5p inhibitor treatment in RAW264.7 cells. (b, d) The levels of Nrf2 and HO-1 proteins were assessed by western blotting after miR-155-5p inhibitor treatment in primary peritoneal macrophage cells. Data are shown as mean ± SD of three independent experiments. NC: negative control. ∗*p* < 0.05, ∗∗*p* < 0.01. ns: nonsignificant.

**Figure 7:**
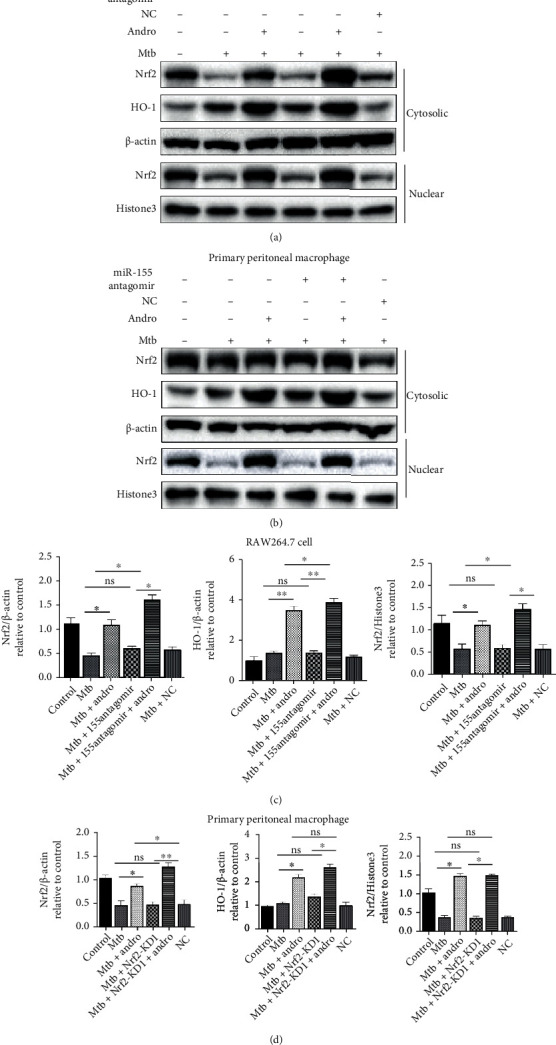
miR-155-5p antagomir upregulates the expression of Nrf2 and HO-1 in Mtb-infected macrophages. (a, c) The expressions of Nrf2 and HO-1 were analyzed by western blotting after miR-155-5p antagomir treatment in RAW264.7 cells. (b, d) The levels of Nrf2 and HO-1 proteins were assessed by western blotting after miR-155-5p antagomir treatment in primary peritoneal macrophage cells. Data are shown as mean ± SD of three independent experiments. NC: negative control. ∗*p* < 0.05, ∗∗*p* < 0.01. ns: nonsignificant.

Then, these observations were validated by transfecting miR-155-5p inhibitor or antagomir into Mtb-infected macrophages and evaluating Nrf2 and HO-1 expression. The results revealed that the inhibition of miR-155-5p resulted in little change in the translocation of Nrf2 but with a significant increase in cytoplasmic HO-1. However, when miR-155-5p inhibitor-treated cells were also exposed to Andro, we noted an increased translocation of Nrf2 compared with Andro alone, although these outcomes were more obvious in RAW264.7 cells (Figures 6(a)–6(d) and 7(a)–(d)). These findings reveal that Andro activates and subsequently promotes Nrf2 translocation into the nucleus by inhibiting miR-155-5p.

### 3.4. Andro Ameliorates Pyroptosis by Activating Nrf2/HO-1 Pathway in Mtb-Infected Macrophages

**Figure 8:**
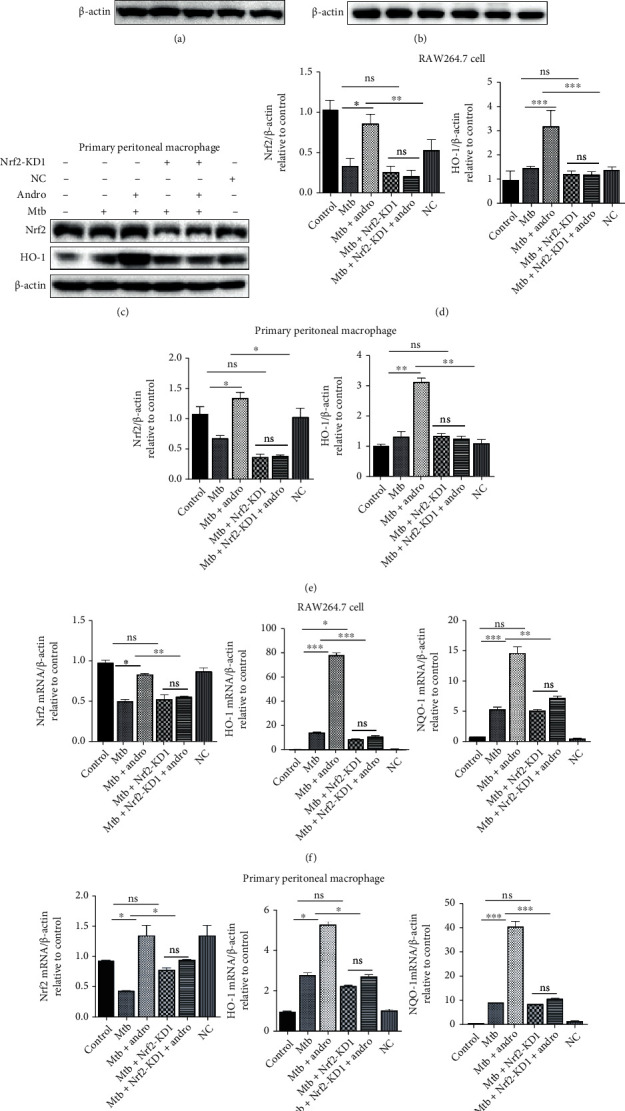
siNrf2 can inhibit the antioxidant effect of Andro in macrophages infected with Mtb. (a) Transfection efficiency of siRNAs was measured by western blotting analysis in RAW264.7 cells. (b, d) The levels of Nrf2 and HO-1 proteins were measured by western blotting after siNrf2 in RAW264.7 cells. (c, e) The levels of Nrf2 and HO-1 proteins were measured by western blotting after siNrf2 in primary peritoneal macrophage cells. (f, g) The levels of Nrf2, HO-1, and NQO-1 mRNAs were measured by RT-PCR after siNrf2 in RAW264.7 cells and primary peritoneal macrophage cells, respectively. Data are shown as mean ± SD of three independent experiments. NC: negative control. ∗*p* < 0.05, ∗∗*p* < 0.01, ∗∗∗*p* < 0.001. ns: nonsignificant.

**Figure 9:**
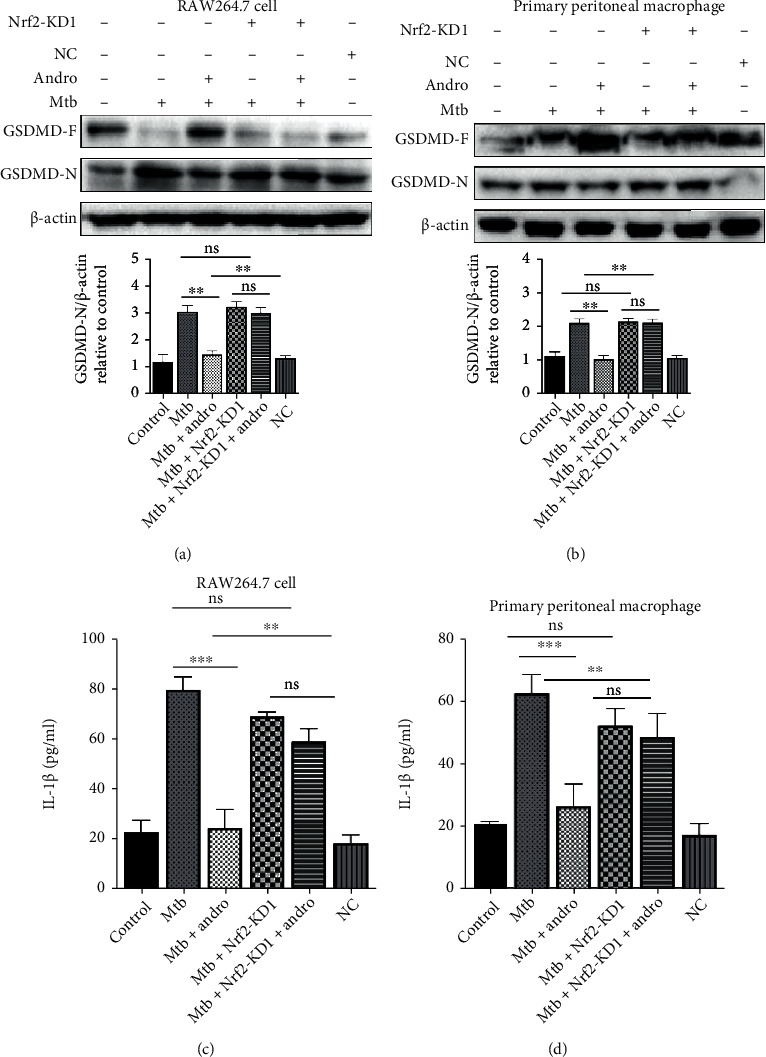
siNrf2 can eliminate the effect of Andro on inhibiting pyroptosis in Mtb-infected macrophages. (a, b) The expressions of GSDMD-N were analyzed by western blotting after siNrf2 in RAW264.7 cells and primary peritoneal macrophage cells, respectively. (c, d) Release of IL-*β* was assessed by ELISA measurement after siNrf2 in RAW264.7 cells and primary peritoneal macrophage cells, respectively. Data are shown as mean ± SD of three independent experiments. NC: negative control. ∗∗*p* < 0.01, ∗∗∗*p* < 0.001. ns: nonsignificant.

Our data support the hypothesis that Andro inhibits miR-155-5p expression activating the Nrf2/HO-1 pathway and that this compound ameliorates pyroptosis in Mtb-infected macrophages. However, it is not clear whether Andro inhibits pyroptosis via activation of the Nrf2/HO-1 pathway. Here, we used siNrf2 to knock down Nrf2 expression and then assessed the level of GSMDM-N. We used three siRNAs to knock down Nrf2 and noted that KD1 had the best effect (Figure 8(a)). Thus, we used KD1 as the siNrf2 in our downstream evaluations. Our results demonstrated that HO-1 was decreased following treatment with siNrf2 and that this could not be rescued by the addition of Andro (Figures 8(b)–8(e)). Similar results also were found in mRNAs levels suggesting that Andro-mediated inhibition of oxidative stress depends on Nrf2 (Figures 8(f) and 8(g)). In order to verify whether Andro treatment suppressed pyroptosis in Mtb-infected macrophages, we evaluated GSDMD-N production following the addition of siNrf2. These results demonstrated that when Nrf2 was knocked down, GSDMD-N was upregulated in Mtb-infected macrophages and that this increase could not be reversed by Andro treatment (Figures 9(a) and 9(b)). Similar results were observed in IL-1*β* level (Figures 9(c) and 9(d)). These results revealed that Andro inhibits pyroptosis in Mtb-infected macrophages through the activation of the Nrf2/HO-1 pathway.

### 3.5. Andro Inhibits Pyroptosis through Its Interactions with the Nrf2/TXNIP/NLRP3 Axis in Mtb-Infected Macrophages

**Figure 10:**
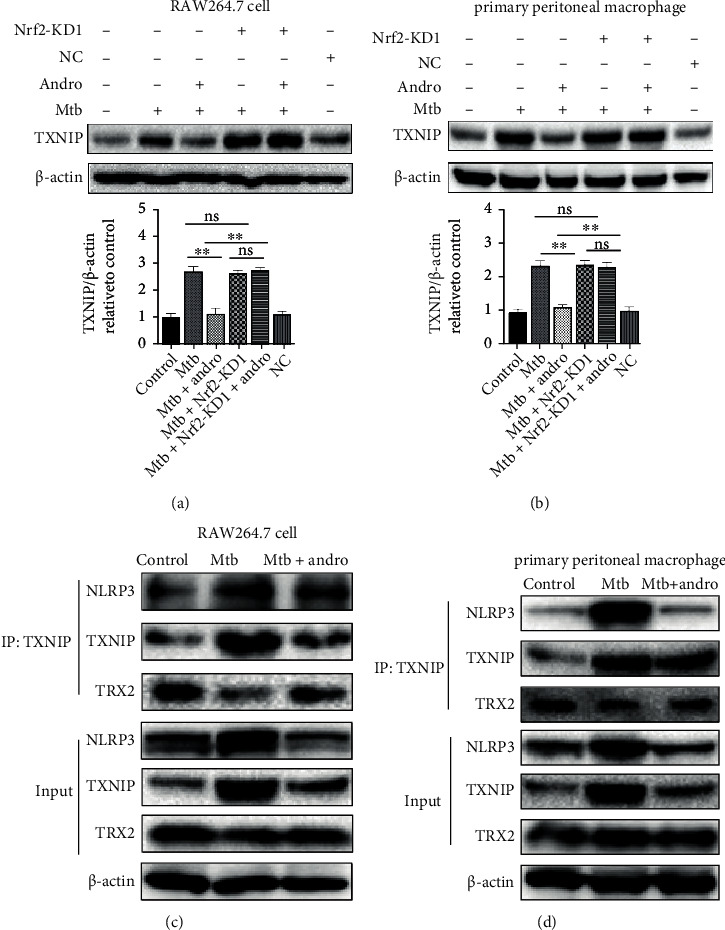
siNrf2 could abolish the effect of Andro on inhibiting the NLRP3 inflammasome activation. (a, b) Levels of TXNIP protein were analyzed by western blotting after siNrf2 in RAW264.7 cells and primary peritoneal macrophage cells, respectively. (c, d) TXNIP or NLRP3 immunoprecipitated from RAW264.7 cells and primary peritoneal macrophage cells were immunoblotted for TXNIP and re-blotted for NLRP3, TXNIP, and TRX2, respectively. Data are shown as mean ± SD of three independent experiments. NC: negative control. ∗*p* < 0.05, ∗∗*p* < 0.01. ns: nonsignificant.

To further study the underlying connections between Nrf2 and the NLRP3 inflammasome, we focused on the effects of these treatments on thioredoxin interacting protein (TXNIP). TXNIP is a member of the thioredoxin (TRX) system and an endogenous regulator of redox/glucose-induced stress and inflammation [56, 57]. TXNIP usually combines with TRX2, but excessive ROS can induce their dissociation allowing free TXNIP to combine with NLRP3 activating it and inducing an excessive inflammatory response [56, 58, 59]. We then investigated whether TXNIP acts as a mediator between Nrf2 and NLRP3 using siNrf2. The results demonstrated that TXNIP was upregulated in Mtb-infected macrophages but was significantly reduced in Andro-treated cells, but that this reduction was inhibited by the addition of siNrf2 (Figures 10(a) and 10(b)). These results indicated that Andro suppresses the expression of TXNIP and that this is dependent on Nrf2.

**Figure 11:**
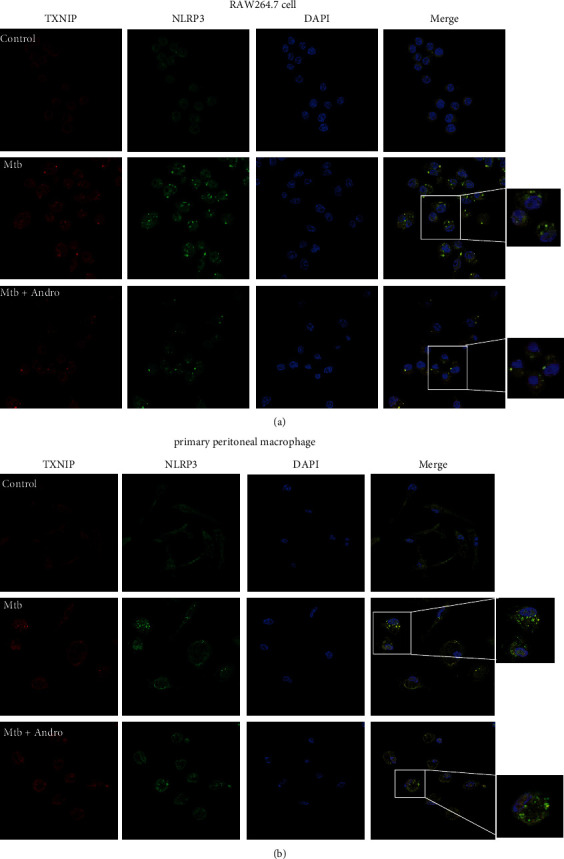
Andro reduces the colocalization of TXNIP and NLRP3 in Mtb-infected macrophages. (a, b) Confocal microscopy of RAW264.7 cells and primary peritoneal macrophage cells with different treatments immunostained with anti-TXNIP (red), anti-NLRP3 (green), and DAPI (blue), respectively. Data are shown as mean ± SD of three independent experiments.

To define whether the effect of Andro on the NLRP3 inflammasome was mediated by the upregulation of Nrf2, coimmunoprecipitation was used to analyze the interaction between TXNIP and TRX2. The interaction was reduced following Mtb infection and was restored following the addition of Andro (Figures 10(c) and 10(d)). We then investigated the interaction between TXNIP and NLRP3 by coimmunoprecipitation and immunofluorescence to determine whether the increased TXNIP expression could lead to NLRP3 inflammasome activation. These results showed that the interaction between TXNIP and the NLRP3 inflammasome increased in Mtb-infected macrophages and that the addition of Andro attenuated this interaction (Figures 10(c) and 10(d)). Confocal imaging also demonstrated that Mtb infection increased the colocalization of NLRP3 and TXNIP, while Andro treatment significantly downregulated their expressions and reduced their colocalization (Figure 11). These results revealed that Andro significantly attenuates pyroptosis via its interactions with the Nrf2/TXNIP/NLRP3 axis in Mtb-infected macrophages.

## 4. Discussion

Current research suggests that the primary cause of death in most TB patients is not their bacterial infection, but rather the tissue damage resulting from their excessive inflammatory response to this infection. Therefore, effective treatment of TB patients is likely to require both elimination of the Mtb and prevention of this inflammatory response. HDT is based on the concept of balancing the host's immune system and may offer a novel approach for the discovery of new anti-TB therapies.

Pyroptosis is a recently described type of cell death, which, unlike apoptosis, may induce excessive inflammatory responses [60]. Studies have reported that Mtb infection can induce macrophage pyroptosis, which in turn induces the release of proinflammatory factors and aggravates tissue damage. In addition, pyroptosis in Mtb-infected macrophages can result in the systemic release of Mtb and increased infection [10].

Andro, an active ingredient extracted from *Andrographis paniculata*, has been reported to have anti-inflammatory and antitumor effects [61, 62]. Our previous study found that this compound can suppress NLRP3 inflammasome activation through the NF-*κ*B signaling pathway, thereby inhibiting the excessive inflammatory response induced by Mtb infection [42]. Mtb infection can also induce pyroptosis and excessive inflammatory responses in macrophages [63], and Andro has a known anti-inflammatory effect. Thus, we hypothesized that Andro might be able to prevent excessive inflammatory responses by inhibiting the pyroptosis in Mtb-infected macrophages. Here, we used western blotting to assess GSDMD-N and PI staining to prove that Andro treatment inhibits pyroptosis in Mtb-infected macrophages. However, due to limited conditions, we were unable to directly observe this phenomenon using electron microscopy. We hope to address this aspect in the future.

The occurrence of pyroptosis is accompanied by many factors, including the production of pro-inflammatory miRNAs and the activation of the inflammasomes [64, 65]. Based on this, we also analyzed these factors to identify the specific mechanism underlying Andro-mediated inhibition of pyroptosis. The previous studies have revealed that miR-155-5p has a strong proinflammatory effect in Mtb infection [66]. In the present study, we evaluated its association with pyroptosis. These results demonstrated that Andro treatment inhibits macrophage pyroptosis by downregulating miR-155-5p.

Given that most miRNAs could exert their effect by promoting the degradation or inhibiting the translation of their target genes, we identified the target genes for miR-155-5p. Our evaluations identified the antioxidant gene Nrf2 as one of the targets of miR-155-5p, and Nrf2 activation is also a known factor in the inhibition of excessive inflammation [34, 36, 67], we evaluated its effects on pyroptosis and whether this might be the mechanism underlying Andro-mediated inhibition of excessive inflammation. Our results revealed that Nrf2 is the target gene of miR-155-5p, and that Andro treatment upregulated the expression of Nrf2 by downregulating miR-155-5p, thereby inhibiting pyroptosis in Mtb-infected macrophages.

**Figure 12:**
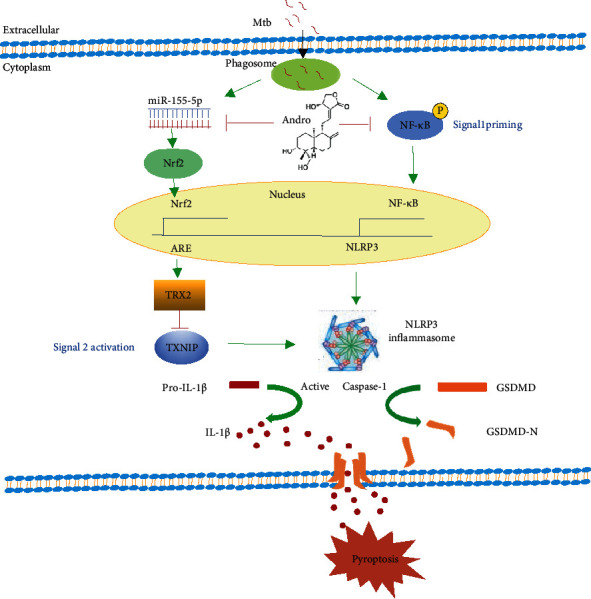
Illustration of Andro suppressing pyroptosis in Mtb-infected macrophages. Previous work has demonstrated that Andro inhibits activation of NLRP3 inflammasome through NF-*κ*B pathway [42]. Here, we revealed that Andro also attenuates pyroptosis in Mtb-infected macrophages via the miR-155-5p/Nrf2 axis.

After activation, Nrf2 is translocated to the nucleus where it promotes the expression of several antioxidant genes, including TRX2, HO-1, and NQO-1 [68, 69]. Under normal conditions, TRX2 combines with TXNIP, but oxidative stress induces their dissociation and allows free TXNIP to combine with the NLRP3 inflammasome, activating the latter and eventually promoting the inflammatory response [70]. Studies have shown that activation of the NLRP3 inflammasome induces the release of various pro-inflammatory factors, which eventually leads to pyroptosis [71]. Our results demonstrated that Andro treatment inhibits NLRP3 inflammasome activation by suppressing the interaction between TXNIP and NLRP3. Thus, our data revealed that Andro likely attenuates pyroptosis in Mtb-infected macrophages via the miR-155-5p/Nrf2 axis (Figure 12).

Of course, there are still some shortcomings in this study. Firstly, we did not use Andro alone to treat normal macrophages to evaluate whether the drug had an effect on factors such as Nrf2, cytokines, and miRNAs. Therefore, we will pay attention to these phenomena in future studies. Besides, we used H37Ra to infect RAW264.7 cell and mouse primary peritoneal macrophages as an in vitro infection model. H37Ra strain is an avirulent strain and has a similar genetic background to H37Rv strain, but it is easier to operate and safer [72, 73]. In the future study, we will evaluate the potential of Andro as an adjuvant therapy against TB in animal models of H37Rv infection.

## Data Availability

The data used to support the findings of this study are available from the corresponding author upon request.
